# 11 million days of longitudinal Fitbit data reveal novel future health insights

**DOI:** 10.1038/s41467-026-78529-2

**Published:** 2026-10-14

**Authors:** Evelynne S. Fulda, Bennett J. Waxse, Slavina B. Goleva, Tam C. Tran, Henry J. Taylor, Caitlin P. Bailey, Dana L. Wolff-Hughes, Huan Mo, Chenjie Zeng, Jacob M. Keaton, Tracey M. Ferrara, Anya Topiwala, Aiden Doherty, Joshua C. Denny

**Affiliations:** 1 Precision Health Informatics Section, National Human Genome Research Institute, National Institutes of Health, Bethesda, MD USA; 2 Big Data Institute, Li Ka Shing Centre for Health Information and Discovery, University of Oxford, Oxford, UK; 3 Nuffield Department of Population Health, University of Oxford, Oxford, UK; 4 National Institute of Allergy and Infectious Diseases, National Institutes of Health, Bethesda, MD USA; 5 British Heart Foundation Cardiovascular Epidemiology Unit, Department of Public Health and Primary Care, University of Cambridge, Cambridge, UK; 6 Heart and Lung Research Institute, University of Cambridge, Cambridge, UK; 7 National Cancer Institute, National Institutes of Health, Bethesda, MD USA; 8 All of Us Research Program, Office of the Director, National Institutes of Health, Bethesda, MD USA

**Keywords:** Diseases, Risk factors

## Abstract

Wearable devices offer a scalable, objective measurement of physical activity, but most studies reduce these data to a single metric over 7-days. Here, we compare wearable-derived phenotyping approaches to understand their impact on activity-disease associations, analyzing 11 million days of Fitbit data from 29,303 *All of Us* Research Program participants. We derive four daily activity metrics (step count, peak 1-min cadence, peak 30-min cadence, and heart rate per step) across five time-windows (1-day, 1-week, 1-month, 6-months, 1-year), then run phenome-wide analyses across > 700 incident and >1,400 prevalent disease outcomes. Though all phenotypes are highly correlated, longer measurement windows yield stronger, more stable associations: 1-year step count was associated with 389 prevalent and 61 incident outcomes (versus 271 and 33 for 1-day step count) after Bonferroni-correction, including novel links to chronic pain syndrome and SARS-CoV-2. Differences between prevalent and incident associations suggest that activity metrics may act as both early markers of disease and risk factors. These findings highlight how large-scale, longitudinal wearable data can advance understanding of health and disease.

## Abstract (web summary)

Researchers linked four Fitbit-derived activity measures, from step count to cadence, to over 400 diseases in 29,303 adults, revealing new associations with conditions including chronic pain and autoimmune disorders.

## Introduction

The World Health Organization estimates that 4 to 5 million deaths per year are attributable to insufficient physical activity^1^. The growing availability of continuous activity monitoring through wearable devices presents a powerful opportunity to understand associations of physical activity, genetics, and other lifestyle factors on diverse health outcomes. Further, it necessitates the need for robust methods to utilize this data for disease detection and risk assessment.

Association analyses tend to use either self-report questionnaires or wearable devices to ascertain physical activity. While surveys are cost-effective to deploy at scale, they have important limitations in the context of physical activity. For example, they are subjective, susceptible to social desirability and recall bias, and tend to only capture intentional activity^2^. In contrast, wearable devices such as wrist-worn accelerometers do not rely on participant recall and may provide a more comprehensive measure of overall activity, capturing both volume and intensity across multiple domains with a single device. However, studies involving wearables must balance resources and the burden on participants with the need to obtain reliable measures of habitual activity. As a result, most wearable studies tend to capture activity over just one week^3^, which may not capture variation in activity and may be confounded by the Hawthorne effect, wherein individuals consciously or unconsciously modify their behavior due to being monitored^4^. These limitations may have an impact on observed associations: while the direction of associations between physical activity and health outcomes tends to be concordant independent of ascertainment method, there are observed discrepancies in effect size when comparing self-reported to device-measured physical activity, and it is unknown if this same discrepancy is observed based on duration of measurement period ^5,6^.

To better understand the determinants and consequences of physical activity and their relationships to health, robust etiologic analyses require accurate and unbiased measurement of habitual activity. Studies indicate that up to 45% of Americans own a wearable device^7^, and as many as 91% own a smartphone capable of measuring physical activity^8^. This widespread access presents an opportunity to leverage data already being passively collected over long time periods. With the growing availability of longitudinal wearable datasets, such as those in the *All of Us* Research Program *(All of Us*)^9^, robust methods for analyzing and integrating wearable-derived data must be developed and evaluated. Using longitudinal wearable device data from *All of Us*, we aimed to 1) compare wearable-derived physical activity metrics averaged across varying time windows and 2) investigate how these metric-time window combinations affect associations with prevalent and incident health outcomes.

## Results

**Fig. 1:**
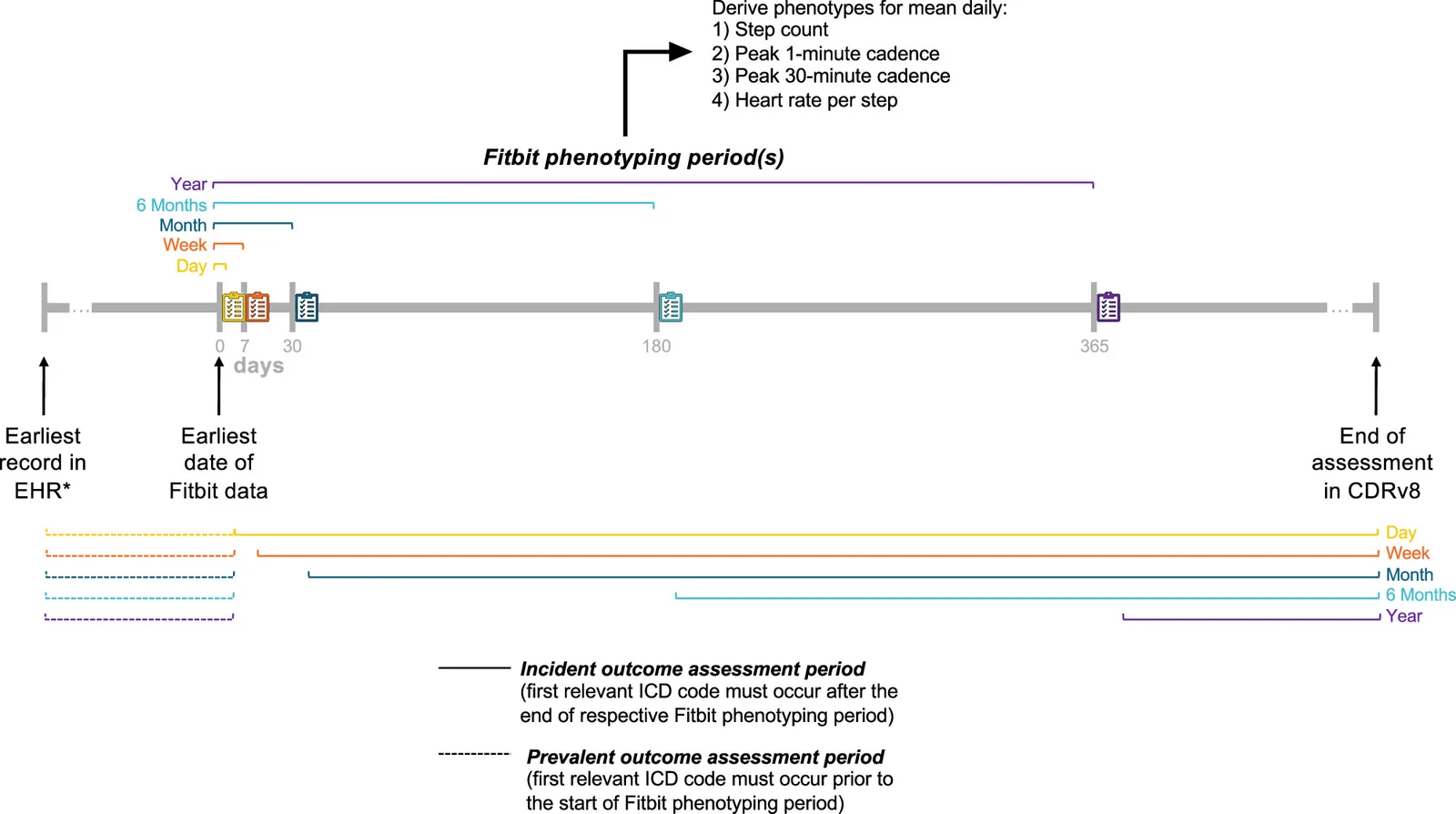
Overview of study design to investigate associations of longitudinal Fitbit activity measurements with prevalent and incident health outcomes among 29,303 participants in *All of Us*. *Earliest record in the EHR could occur at any time, including after the earliest date of Fitbit data. Four phenotypes were derived from minute-level Fitbit physical activity data at five time points (1-day, 1-week, 1-month, 6-months, 1-year). Prevalent disease was defined as the presence of at least two relevant ICD codes on different dates, with the first code occurring prior to the start of the Fitbit phenotyping period. Incident disease was defined as the presence of at least two relevant ICD codes on different dates, with the first code occurring after the end of the respective Fitbit phenotyping period. Abbreviations: Curated data repository version 8 (CDRv8); Electronic health record (EHR); International Classification of Diseases (ICD). Description of Fitbit-derived phenotypes in Supplementary Table 1.

Among 29,303 participants with both Fitbit and electronic health record (EHR) data enrolled in *All of Us* (flow diagram shown in Supplementary Fig. 1), we derived four activity metrics (daily step count, peak 1-min cadence, peak 30-min cadence, and heart rate per step [DHRPS]) across five time windows (1-day, 1-year, 1-month, 6-months, and 1-year) from longitudinal wearable device data, yielding 20 activity phenotypes. We conducted a phenome-wide analysis of associations between each physical activity metric-time window combination and health outcomes, using logistic regression for prevalent cases and Cox regression for incident cases, with cases defined from primary and secondary care EHRs using International Classification of Diseases (ICD) codes aggregated into phecodes^10–12^ (Fig. 1).

### Baseline characteristics

Baseline participant characteristics are presented in Table 1 and median (IQR) values for all twenty phenotypes are presented in Supplementary Table 2. The median age at the start of Fitbit assessment was 57.3 (interquartile range [IQR]: 42.3, 69.3) years. Over two-thirds of participants were female (69%), the majority were current alcohol drinkers (81%), and more than one third of participants were current/past smokers (38%). The 1-year median (IQR) daily step count was 7040 (4880, 9520), and around 46% of participants had at least one year of valid Fitbit data. Compared with the broader EHR sample, individuals with Fitbit data included a higher proportion of females, fewer participants identifying as Hispanic/Latino or Black, and more frequent alcohol consumers. However, they did not differ by age at the *All of Us* censor date (October 1, 2023) or by body mass index (BMI). Participants with Fitbit data either already owned a device and opted to share their data (Bring Your Own Device [BYOD] cohort) or were provided a device by *All of Us* (Wearables Enhancing *All of Us* Research [WEAR]). WEAR participants had a lower proportion of females and participants identifying as White, as well as a higher proportion of individuals in the lowest income group (<$35k/year), compared with BYOD participants. WEAR participants also demonstrated a lower median step count, and fewer participants had at least one year of Fitbit data available.

**Table 1:** Baseline characteristics among participants with EHR data

	Analysis cohort			
	WEAR participants (N = 15,846)	BYOD participants (N = 13,457)	WEAR and BYOD participants (N = 29,303)	All participants with EHR data (N = 351,212)
**Age at** ***All of Us*** **censor date**	58.3 [42.3, 70.3]	57.3 [42.3, 68.3]	57.3 [42.3, 69.3]	58.3 [42.3, 69.3]
**Age at start of Fitbit assessment**	57.5 [41.0, 70.0]	52.8 [38.5, 63.6]	55.0 [40.0, 67.1]	-
**Sex at birth**				
Female	10485 (66.2%)	9687 (72.0%)	20172 (68.8%)	219,257 (62.4%)
**Race/ethnicity**				
Black	1651 (10.4%)	648 (4.8%)	2299 (7.8%)	57,849 (16.5%)
Hispanic	1412 (8.9%)	448 (3.3%)	1860 (6.3%)	53,335 (15.2%)
Other	3366 (21.2%)	1182 (8.8%)	4548 (15.5%)	43,690 (12.4%)
White	9417 (59.4%)	11179 (83.1%)	20596 (70.3%)	196,338 (55.9%)
**Alcohol consumption**				
Never drinker	655 (4.1%)	391 (2.9%)	1046 (3.6%)	35,404 (10.1%)
Past drinker	2948 (18.6%)	1592 (11.8%)	4540 (15.5%)	60,602 (17.3%)
Current, monthly	8397 (53.0%)	7281 (54.1%)	15678 (53.5%)	172,869 (49.2%)
Current, weekly	3846 (24.3%)	4193 (31.2%)	8039 (27.4%)	82,337 (23.4%)
**Smoking history**				
Never smoker	9378 (59.2%)	8836 (65.7%)	18214 (62.2%)	208,570 (59.4%)
**Annual income**				
<$35k	4885 (30.8%)	1690 (12.6%)	6575 (22.4%)	107,074 (30.5%)
$35-$75k	4056 (25.6%)	3319 (24.7%)	7375 (25.2%)	66,646 (19.0%)
$75-$100k	3965 (25.0%)	4825 (35.9%)	8790 (30.0%)	66,869 (19.0%)
>$150k	1793 (11.3%)	2825 (21.0%)	4618 (15.8%)	40,887 (11.6%)
Prefer not to answer	1147 (7.2%)	798 (5.9%)	1945 (6.6%)	69,736 (19.9%)
**Body mass index**^**a**^				
Underweight	208 (1.3%)	98 (0.7%)	306 (1.0%)	4,579 (1.3%)
Healthy weight	4098 (25.9%)	3468 (25.8%)	7566 (25.8%)	90,399 (25.7%)
Overweight	4624 (29.2%)	4264 (31.7%)	8888 (30.3%)	106,818 (30.4%)
Obesity	6916 (43.6%)	5627 (41.8%)	12543 (42.8%)	149,416 (42.5%)
**Year – step count**	6330 [4180, 8910]	7780 [5750, 10100]	7040 [4880, 9520]	-
**Year – peak 1-min cadence**	103 [91.6, 112]	110 [100, 119]	106 [95.5, 116]	-
**Year – peak 30-min cadence**	70.0 [54.6, 85.5]	80.4 [66.8, 94.0]	75.0 [60.0, 89.9]	-
**Year – heart rate per step**	0.0149 [0.0102, 0.0236]	0.0119 [0.00874, 0.0169]	0.0133 [0.00939, 0.0201]	-
**Participants with at least one year of Fitbit data**	2955 (18.6%)	10638 (79.1%)	13593 (46.4%)	-

^a^World Health Organization (WHO) classification – Underweight ( < 18.5 kg/m^2^); Healthy weight ( ≥ 18.5 kg/m^2^ - <25 kg/m^2^); Overweight (>=25 kg/m^2^ - <30 kg/m^2^); Obesity ( ≥ 30 kg/m^2^)

Data are presented as median [IQR] for continuous variables and n (%) for categorical variables. *BYOD* Bring Your Own Device, *DHRPS* Daily heart rate per step, *EHR* Electronic health record, *WEAR* Wearable Enhancing All of Us Research. Description of variables listed in Supplementary Table 1.

### Comparison of physical activity phenotypes

Across all twenty metric-time window combinations, absolute Pearson correlation coefficients ( | r | ) were generally high (median [IQR] |r | : 0.71 [0.64–0.80]; Supplementary Fig. 2a). Daily heart rate per step (DHRPS) was inversely associated with the other device-measured metrics. All grouped comparisons are shown in Supplementary Table 3.

A similarity network demonstrates the relative similarity of metrics across time windows (Supplementary Fig. 2b).

Sankey diagrams that grouped participants in quartiles for each phenotype showed that participants tend to be in the same quartile of a metric regardless of time window (Supplementary Fig. 3).

Reliability of measurements increased with more days of wear for step count (range of ICCs: 0.68 for 1-day to 0.90 for 60-days), peak 1-min cadence (0.59 for 1-day to 0.89 for 60-days), peak 30-min cadence (0.68 for 1-day to 0.90 for 36-days), and DHRPS (0.71 for 1-day to 0.90 for 58-days; Supplementary Fig. 2c). For all four metrics there was a plateau of ICC around 30-days.

### Phenome-wide associations between physical activity phenotypes and both prevalent and incident outcomes

**Fig. 2:**
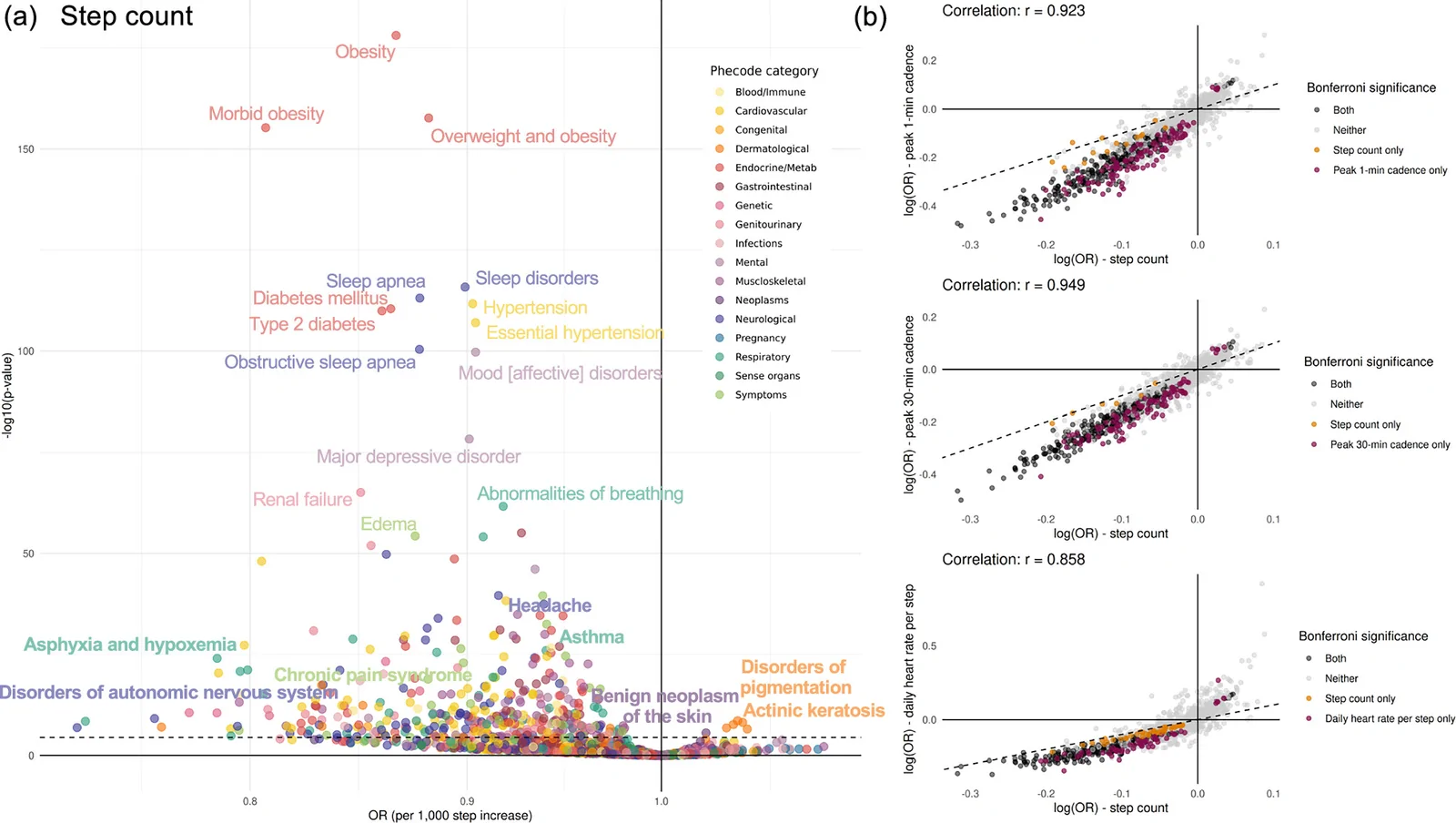
Prevalent phenome-wide associations with physical activity from one-year of wear. **a** Volcano plot shows associations between year step count and all phecodes. The horizontal dotted line denotes the Bonferroni-corrected significance threshold (α = 0.05/1402). P-values are two-sided. Bolded phecodes are those that have not been shown in prior PheWAS for physical activity. **b** Scatter plots show the association between log odds ratios for step count and the three other physical activity metrics (peak 1-minute cadence, peak 30-minute cadence, and inversed daily heart rate per step) across phecodes. Log(OR) were estimated from year metrics. Dashed diagonal lines indicate equality of effects between metrics. Points are colored by Bonferroni-corrected significance (α = 0.05/1402).

Participants had a median (IQR) of 8.0 (5.2–13.1) years of EHR data available prior to the start of the Fitbit phenotyping period, which was used for ascertainment of prevalent disease. For incident disease analyses, the median (IQR) duration of EHR follow-up after completion of the Fitbit phenotyping period was 1.9 (0.2–4.8) years for the 1-day window, 2.0 (0.2–4.8) years for the 1-week window, 2.2 (0.2–4.9) years for the 1-month window, 3.6 (1.7–5.5) years for the 6-month window, and 3.4 (1.7–5.2) years for the 1-year window. PheWAS analyses identified widespread associations between physical activity phenotypes and both prevalent (year analysis *n* = 389, *n* = 480, *n* = 483, *n* = 402 for step count, peak 1-min cadence, peak 30-min cadence, and DHRPS, respectively) and incident (year analysis *n* = 61, *n* = 97, *n* = 86, *n* = 83 for step count, peak 1-min cadence, peak 30-min cadence, and DHRPS, respectively) Bonferroni-significant outcomes. Patterns of association were largely consistent across metrics (Fig. 2b); therefore, subsequent results focus on step count, a widely interpretable measure of physical activity (Fig. 2a). PheWAS results for all metric-time window combinations are presented in Supplementary Data 1; Manhattan plots are shown in Supplementary Figs. 4, 5.

We replicated 232 out of 379 (61%) associations reported in prior physical activity PheWAS^13–19^ and identified 310 new incident and/or prevalent physical activity-disease associations, including with benign neoplasm of the skin, cardiomyopathy, and systemic lupus erythematosus (Supplementary Fig. 6). Associations we did not replicate included ulcerative colitis and pleurisy.

**Fig. 3:**
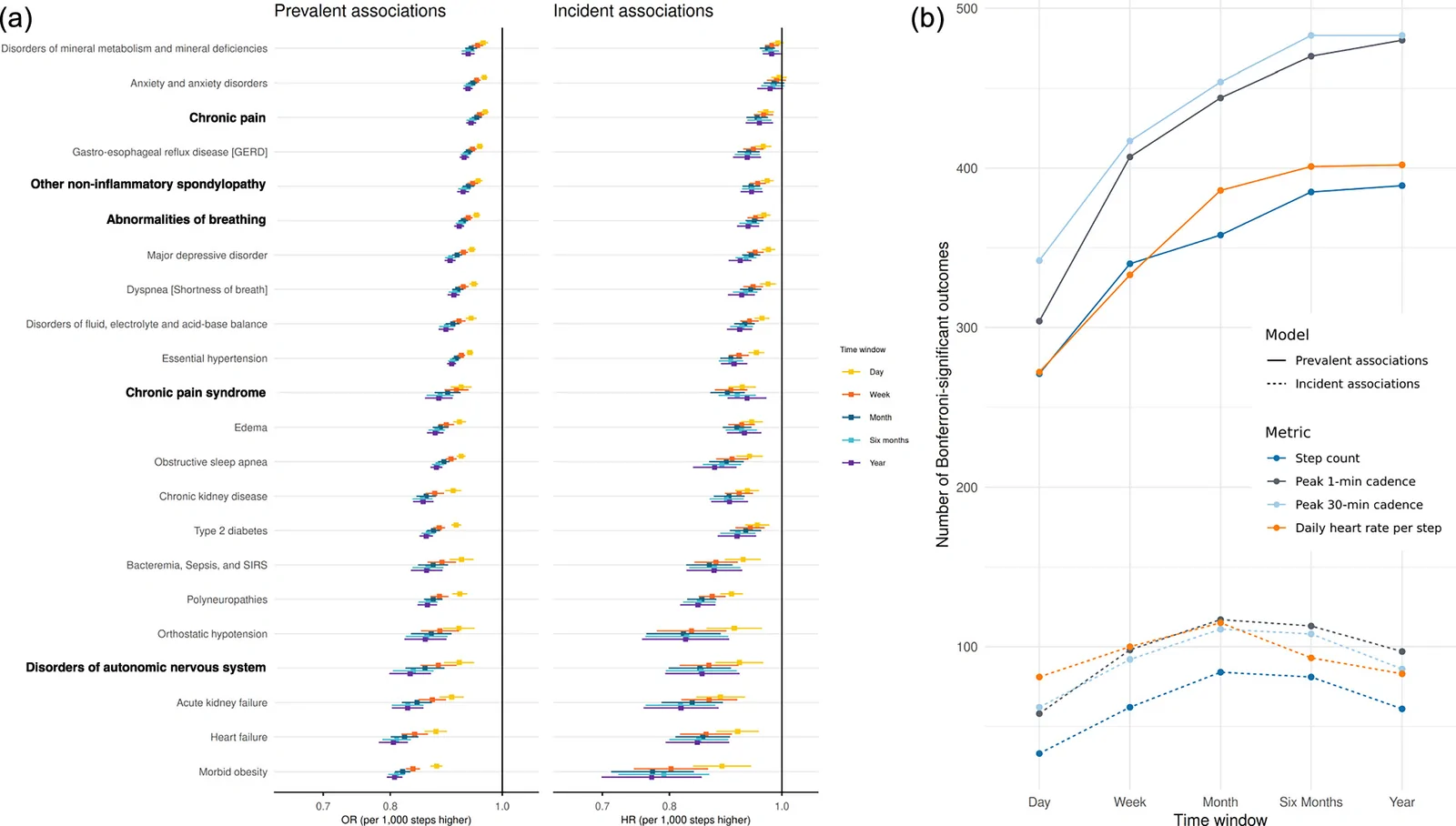
Effect of time window on physical activity-disease associations. **a** Associations between daily step count and selected Bonferroni-significant clinical outcomes based on a PheWAS across five time windows (1-day, 1-week, 1-month, 6-months, 1-year) using logistic (prevalent cases) and Cox (incident cases) regression. Here we show associations with the top 20 unique phecodes. Risk estimates are scaled per 1000 additional steps. Forest plots display odds ratios (ORs) and hazard ratios (HRs) with 95% confidence intervals, color-coded by time window. Regression models adjusted for age at the start of the Fitbit phenotyping period, sex at birth, race, ethnicity, alcohol consumption, smoking history, income, and WEAR/BYOD status. Bolded phecodes are those that have not been shown in prior PheWAS for physical activity. **b** Lines show the number of phenotypes meeting Bonferroni significance for each physical activity metric across different follow-up periods. Colors distinguish measures (Step count, Peak 1-min cadence, Peak 30-min cadence, Daily heart rate per step), while line type distinguishes models (solid = Logistic regression, dashed = Cox regression).

Associations were directionally consistent across all time windows within a metric, although the strength of association diminished as the number of days included in the phenotype decreased (Fig. 3a). Pairwise z-score comparisons of regression coefficients across time windows indicated significant differences for some windows but not others (Supplementary Fig. 7). For example, the risk of disorders of the autonomic nervous system per 1000 steps higher from the 1-year phenotype was HR (95% CI): 0.77 (0.70–0.85). The same comparison from the 1-day phenotype was 0.89 (0.84–0.94) with evidence for a pairwise difference (z = −2.4, *p* = 0.02). Results for all metrics are shown in Supplementary Fig. 7.

The number of Bonferroni-significant associations generally increased with longer time windows across all four metrics (Fig. 3b), with a levelling off around one month for incident analyses, although this may be partially due to shorter follow-up for 6-month and 1-year phenotypes and therefore reduced power. For example, 271 phecodes were significant in the step count 1-day prevalent analysis compared to 389 in the step count 1-year prevalent analysis. However, the sets of significant associations were not fully overlapping: some outcomes identified at shorter windows were not significant at longer windows, and vice versa (Supplementary Fig. 8 and Supplementary Data 2). Overlap increased as the time windows lengthened, for example, with greater concordance between year and six-month analyses and less between day and year.

**Fig. 4:**
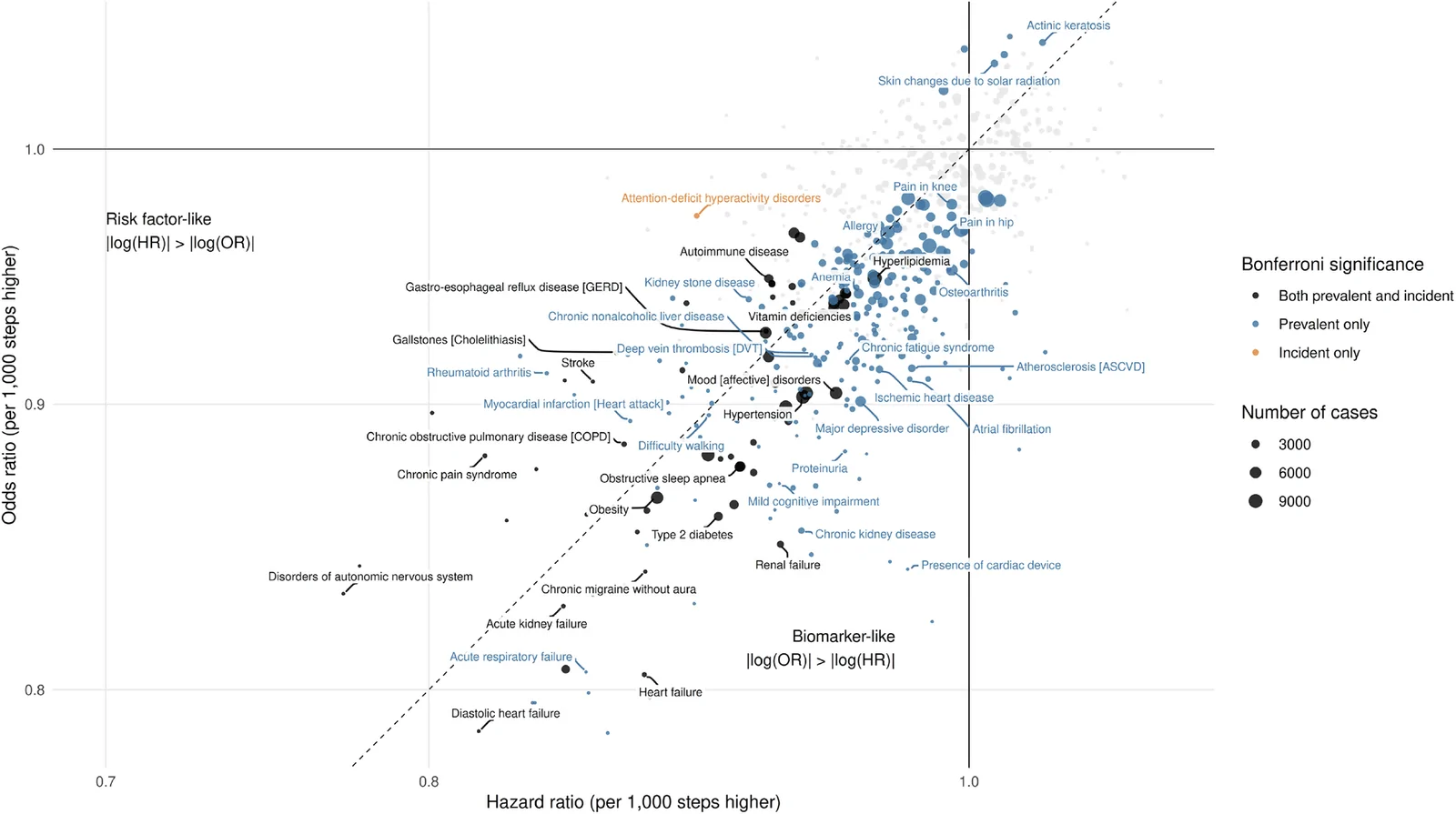
Comparison of prevalent and incident associations with step count. Each point represents a phecode analyzed in both logistic regression (prevalent cases, y-axis) and Cox regression (incident cases, x-axis), with effect sizes shown as odds ratios (OR) and hazard ratios (HR) per 1000 steps higher from the 1-year Fitbit assessment. Points are colored by whether significance occurred only in the prevalent analysis (blue), incident analysis (orange), or both (black), with size denoting the number of cases.

Comparisons of modeling approaches indicated that associations were generally stronger and confidence intervals narrower in analyses of prevalent disease compared with incident disease Cox models. For step count, 60 associations were significant in both prevalent and incident analyses, 252 were significant only in the prevalent analysis, and one (attention-deficit hyperactivity disorder) was significant only in the incident analysis (Fig. 4). The other three metrics are shown in Supplementary Fig. 9. While the larger case numbers in the prevalent analyses likely contribute to these differences, differences in associations or strengths of association in the two analyses may also suggest that these metrics function as early markers of disease (stronger in prevalent analysis), risk factors (stronger in incident analysis), or both. For instance, health outcomes like pain in the knee, hip, or joint, and rheumatoid arthritis were significantly inversely associated with step count only in the prevalent analysis, which may indicate that walking pace does not increase the risk of disease-related pain, but rather that experiencing pain influences an individual’s activity levels.

Findings were generally consistent in four sensitivity analyses. Compared to the main analysis, after adjusting for BMI or excluding events in the first three years, associations were slightly attenuated (Supplementary Fig. 10).

## Discussion

This analysis, leveraging ~11.3 million person-days of Fitbit measurements from 29,303 participants, yielded three key insights. First, we observed 300+ novel associations compared to prior physical activity PheWAS. Comparisons of prevalent and incident analyses suggest that physical activity metrics may function as both risk factors for some diseases and early markers of existing disease for others. Second, although all time windows yielded informative associations, longer assessment periods were generally associated with greater effect sizes for disease risk, with differences across time windows confirmed statistically using z-score comparisons. Counts of statistically significant associations for each activity phenotype broadly plateaued at around 6 months. Third, Fitbit-derived activity phenotypes were highly correlated across metrics and time windows.

While prior studies have looked at phenome-wide associations with physical activity^13–19^, our analysis both confirmed known relationships (e.g., obesity, sleep apnea, type 2 diabetes) and revealed other, potentially novel and more detailed associations, including with autoimmune disorders (lupus, demyelinating diseases of the central nervous system, diffuse diseases of connective tissue), chronic pain syndrome, and bipolar disorder.

We observed differences in associations between prevalent and incident disease models across all four metrics. Associations with larger effect sizes in prevalent analyses suggest that these measures may act as early markers of existing disease, for example, capturing reduced mobility or exercise capacity among individuals already affected by disease. By contrast, stronger associations observed in incident analyses suggest that lower activity levels may precede disease onset, pointing to a potential risk factor role. However, these findings should be interpreted cautiously, as they may also reflect medical coding practices, delayed diagnoses, or treatment-related effects. For example, we observed an association between step count and incident, but not prevalent, ADHD. One possible explanation is that activity levels among individuals diagnosed with ADHD may be altered by medication use or increased awareness following diagnosis. These findings emphasize that step count, cadence, and daily heart rate per step capture complementary aspects of activity and physiological function, and that considering both prevalent and incident associations is important for understanding potential bidirectional associations. More broadly, these findings may provide insight into the distinct biological pathways through which activity patterns influence health and vice versa.

Notably, we found that longer observation windows yielded stronger associations with larger effect sizes between activity metrics and health outcomes, a finding that persisted across multiple sensitivity analyses. While these differences may seem modest, they have important implications for translation. For example, every 1000 additional steps in the 1-year phenotype was associated with an 18% lower risk of chronic pain syndrome (HR = 0.82). To achieve equivalent estimates using the 1-day or 1-week phenotype (HR = 0.89 and HR = 0.87 per 1000 steps, respectively) would require more steps: 1703 steps/day for the 1-day phenotype and 1425 steps/day for the 1-week phenotype. These differences highlight how methodological choices, such as the time window used to define the exposure, can meaningfully influence effect estimates and their interpretation, even among the same population. These findings are consistent with measurement error theory, which holds that extending the measurement window brings the observed value closer to the true underlying construct, thereby reducing random error and minimizing attenuation toward the null^20^. While many observed incident associations were concordant across the time windows within each metric, some activity-outcome associations were only observed at longer time windows. For instance, risk of systemic lupus erythematosus was only Bonferroni-significant at the 6-month and 1-year time windows for both step count and daily heart rate per step, underscoring the potential to miss associations when phenotyping is based on shorter periods. This may be due to long-term activity better reflecting habitual behavior and/or aligning with the fact that many chronic outcomes progress over longer periods. Overall, our results indicate that longer measurement periods yield stronger and more stable associations. However, even brief monitoring windows capture meaningful signals of disease risk, with modest improvements in reliability (ICC) observed when extending data collection from 7 to 30 days for all metrics, and overall similarities in associations with health outcomes; the optimal monitoring period will depend on the health outcome of interest as well as available resources.

The Fitbit-derived metrics were highly reliable as reflected by their ICCs. This finding is consistent with other studies using wearable devices to measure activity^21–23^. In contrast, survey-based methods for assessing physical activity tend to have low reliability^2^. Correlations between the different metrics were also high but lower than within-metric correlations across time windows. This observation suggests that each phenotype may capture distinct physical activity domains, consistent with prior studies^24^. PheWAS results also supported this finding: while there were overlaps in associations with certain outcomes across the four metrics, there were also unique associations. For example, cadence metrics and DHRPS, measures of activity intensity, were more strongly associated with acute respiratory failure and emphysema than step count, a measure of activity duration.

To our knowledge, this is the largest cohort to date with longitudinal wearable device data linked to primary and secondary care EHRs to assess prevalent and incident health outcomes. Leveraging this unique dataset enabled a comprehensive assessment of four physical activity metrics across multiple phenotyping periods in nearly 30,000 participants, followed by robust analyses of disease risk. As many of the individuals in this study still had relatively brief observation periods, we anticipate the power to detect differences in outcomes will grow significantly over time.

Limitations caution interpretation of this study. First, although we leveraged record linkage with a participant’s EHR, we may have missed events if they were not coded correctly or were coded outside of the participant’s linked health record. Several outcomes are also known to have long preclinical and prodromal periods and therefore may not have been officially diagnosed at the time of analysis. Although prospective analyses were performed, the median follow-up period was relatively short for many chronic diseases. Consequently, these findings should be interpreted as evidence of prospective associations or early biomarkers rather than definitive etiological relationships, given the susceptibility to reverse causation, whereby pre-existing subclinical disease may influence physical activity behavior rather than the reverse^25^. Whilst a sensitivity analysis excluding events from the first three years of follow-up yielded broadly consistent findings, longer-term studies will be required to establish temporal and potentially causal pathways. Second, some covariates, such as income, smoking history, and alcohol consumption, were collected at baseline rather than at the onset of Fitbit monitoring and therefore may not reflect participants’ most current status; further, participants contributing Fitbit data may differ from those who do not, and similar differences may exist between BYOD and WEAR participants. Our comparisons suggested that these group differences were relatively small, and all observed imbalances, including the tendency for BYOD participants to contribute longer follow-up, were adjusted for in the association analyses. The observed differences in follow-up primarily reflect the later initiation of the WEAR program and underscore the importance of recruiting diverse cohorts early and sustaining longitudinal follow-up in wearable-based research. Third, step counts were derived from Fitbit devices, which, while widely used and validated, are subject to measurement error that may vary by device model, wear behavior, and activity type. Such error could introduce bias into estimated associations, including attenuation due to regression dilution. Fourth, given the number of assessed outcomes, we evaluated only linear associations, which may miss relevant non-linear relationships.

In summary, Fitbit-derived physical activity phenotypes were reliable, complementary, and associated with a wide range of health outcomes. Our findings confirm known associations while also identifying novel links, and they demonstrate that both the choice of metric and the duration of the observation time window influence the effect size of observed relationships. Longer monitoring periods consistently yielded stronger and more stable associations, though even short-term windows retained consistent associations, making them feasible for studies or clinical settings with limited longitudinal data. Differences between prevalent and incident associations demonstrate the dual role of activity measures as both early markers and risk factors. While more research is needed to replicate these findings in other populations, these findings underscore that wearable devices, currently worn by hundreds of millions of people around the globe, can advance population health research and inform scalable approaches for clinical risk stratification.

## Methods

This analysis used data from the *All of Us* Research Program Controlled Tier dataset. This research complies with all relevant ethical regulations. The *All of Us* Research Program’s protocol and consent process were reviewed and approved by the *All of Us* Institutional Review Board; all participants provided written informed consent. Data use complied with the requirements of the *All of Us* Research Program Data User Code of Conduct.

### Study design and participants

*All of Us* is a US-based longitudinal cohort study with 884,000+ participants as of June 2026. Recruitment and enrollment procedures have been described previously^9^. This analysis used data from the Curated Data Repository version 8 (C2024Q3R8). Participants provided informed consent and completed surveys through a digital platform. Most participants consented to linkage with their EHR data.

### Physical activity assessment

Physical activity was measured using Fitbit devices^26^. Initially, the integration of Fitbit data followed a “Bring Your Own Device” (BYOD) model, wherein enrolled participants who owned a Fitbit device could link their device data, allowing for sharing of data that was collected prior to enrollment. In 2021, *All of Us* launched the Wearables Enhancing *All of Us* Research (WEAR) study with the goal of expanding the number of participants contributing device data and enhancing the representation of individuals with Fitbit data by providing devices to participants from populations that had been less represented in the BYOD cohort.

We focused on four Fitbit-derived metrics (Supplementary Table 1): daily step count, peak 1-min cadence, peak 30-min cadence, and heart rate per step (DHRPS). Step count is the total number of steps in a 24-h day. The cadence metrics are derived by ordering each measure of steps/min in a 24-h day and taking the highest value (peak 1-min cadence) or the average of the 30 highest values (peak 30-min cadence). DHRPS is calculated by dividing the average daily heart rate by daily step count ^14^.

Each metric was averaged at five time windows: 1-day, 1-week, 1-month, 6-months, and 1-year intervals, in relation to the earliest date of valid Fitbit data (Fig. 1). Valid days required ≥16 h of wear time^27^ (based on minutes with a heart rate value) and values within prespecified feasible ranges (step count >100 and <75,000; cadence <210 step/min; DHRPS < 3 standard deviations above the mean; Supplementary Fig. 1); invalid days were excluded. Arithmetic means were calculated from all valid days within each window; for example, if a participant had two valid days of Fitbit data in the 1-week time window, the average of the two was taken. This approach increased sample size and reflects real-world settings in which data collection is often incomplete and wear time varies across individuals.

### Outcome ascertainment

Aggregated EHR data includes inpatient and outpatient data from healthcare provider organizations, including primary care, and participant-mediated patient portal linkages. Disease outcomes were defined using phecodes, manually curated codes that group ICD-9 and ICD-10 codes^10,11^. Phecodes are hierarchical: a parent phecode includes all of its descendants. Each phecode was analyzed as a separate outcome. Most phecodes have no child phecodes, but some do, and a parent phecode’s case definition includes all of its descendant phecodes. Because of this hierarchical structure, a single participant can contribute to multiple related outcomes: for example, a participant who is a case for Type 2 diabetes (phecode: EM_202.2) would also be a case for the parent phecode Diabetes mellitus (phecode: EM_202). Associations across hierarchically related phecodes are therefore not statistically independent, and findings across related conditions should be interpreted as potentially reflecting the same underlying signal at different levels of diagnostic specificity. Our Bonferroni correction for multiple testing remains conservative given this overlap. For each outcome of interest, cases were defined as participants having at least two corresponding ICD codes recorded on separate dates. We included all PheCodes from PhecodeX with at least 50 cases ^12^.

### Covariates

Covariates included age at the start of Fitbit phenotyping, WEAR/BYOD status as well as self-reported sex at birth, race/ethnicity, alcohol consumption, smoking history, and annual income (Supplementary Table 1). All self-reported variables were assessed at enrollment. A sex at birth of male or female was required for the phenome-wide analyses, and therefore participants who were intersex or declined to report were excluded. Responses where participants declined to answer or skipped a question were considered missing. For income, declining to answer was coded as a separate category, as non-response may itself be informative. Three covariates (race/ethnicity, alcohol consumption, smoking status) had low levels of missing data (2%, 4%, 3%, respectively) and were imputed.

### Statistical analysis

Pairwise Pearson correlations (r) were examined among phenotypes across metrics and time windows; for the similarity network, similarity was defined as r². To assess stability across time windows, each metric was binned into quartiles at each time window, and transitions were visualized using Sankey diagrams. Reliability of each metric was evaluated by calculating intraclass correlation coefficients (ICC) between the first valid day of Fitbit data and a randomly selected day at least one week later, repeated ten times; we then incrementally increased the number of days contributing up to 60.

Associations between each metric-time window combination and disease outcomes were evaluated using a phenome-wide (PheWAS) approach, with logistic regression for prevalent cases and Cox proportional hazards models for incident cases (Fig. 1). Cases were participants with at least two relevant ICD codes for each phecode; participants with only one ICD code were excluded for that analysis. For the logistic regression models, only cases with their first ICD code occurring prior to their first date with Fitbit data were included. For the Cox regression models, only cases with their first ICD code occurring after the end of the respective phenotyping time window were included. Follow-up began at first Fitbit use and ended at the first relevant ICD code or at the last EHR date. Analyses used PheTK^28^, and multiple testing was accounted for using a Bonferroni correction (a = 0.05 / # of phecodes). Significant associations were compared with seven prior physical activity PheWAS from large studies^13–19^. Differences in outcome coding prevented direct one-to-one matching across studies; however, this comparison enabled a broad evaluation of the novelty of the present analysis.

Differences in associations across time windows were tested using a z-score:

1 $$z=\frac{{\beta }_{a}-{\beta }_{b}}{\sqrt{{{SE}}_{a}^{2}+{{SE}}_{b}^{2}}}$$

where *a* and *b* are a metric from different time windows. This is a conservative approach as it does not account for the correlations between the time windows; this approach was chosen to reduce Type I error (false positives).

In a hypothesis-generating analysis, to assess whether the strength of the association between a metric and an outcome differed in the prevalent vs. incident analysis, we examined ratios of the odds ratios to hazard ratios from the 1-year phenotypes. We note that these estimates are derived from different models and are not directly comparable in interpretation, but this comparison provides a general sense of relative association strength.

We conducted four sensitivity analyses: (1) additional adjustment for BMI (assessed at the baseline study visit based on measured height and weight and categorized using WHO classifications); (2) including a “washout” period, wherein we excluded the first two weeks of Fitbit data and recalculated all phenotypes^26^; (3) restricting to participants with ≥1 year of Fitbit data; and (4) excluding incident events occurring within the first three years of Fitbit wear.

All analyses were conducted using R, version 4.4.0 (R Core Team, Austria). Results are reported according to the STROBE guidelines^29^. Analysis notebooks are available to any approved *All of Us* controlled tier researchers. A GitHub repository with code can be found at: https://github.com/OxWearables/aou-11-million-days-fitbit (https://doi.org/10.5281/zenodo.22131079).

### Reporting summary

Further information on research design is available in the Nature Portfolio Reporting Summary linked to this article.

## Supplementary information

Supplementary Information

Description of Additional Supplementary Information

Supplementary Data 1

Supplementary Data 2

Reporting Summary

Transparent Peer Review file

## Data Availability

Data were from the *All of Us* Research Program and are available to all approved researchers on the *All of Us* Researcher Workbench. Researchers can register for access at www.researchallofus.org.

## Code availability

All analyses were conducted using R and were performed within the *All of Us* Researcher Workbench. Analysis notebooks are available to any approved *All of Us* controlled tier researchers. A GitHub repository with code can be found at: https://github.com/OxWearables/aou-11-million-days-fitbit (https://doi.org/10.5281/zenodo.22131079).

## References

[1] Health topics: physical activity. World Health Organization. (WHO, 2024).

[2] Shephard, R. J. Limits to the measurement of habitual physical activity by questionnaires. *Br. J. Sports Med.* **37**, 197–206 10.1136/bjsm.37.3.197 (2003). PMC1724653 12782543

[3] Chan, A., Chan, D., Lee, H., Ng, C. C. & Yeo, A. H. L. Reporting adherence, validity and physical activity measures of wearable activity trackers in medical research: a systematic review. *Int. J. Med. Inform.* **160**, 104696 10.1016/j.ijmedinf.2022.104696 (2022). 35121356

[4] Sedgwick, P. & Greenwood, N. Understanding the Hawthorne effect. *BMJ Br. Med. J.* **351**, h4672 10.1136/bmj.h4672 (2015). 26341898

[5] Ekelund, U. et al. Dose-response associations between accelerometry measured physical activity and sedentary time and all cause mortality: systematic review and harmonised meta-analysis. *BMJ* **366**, l4570 10.1136/bmj.l4570 (2019). PMC6699591 31434697

[6] Wasfy, M. M. & Lee, I.-M. Examining the dose–response relationship between physical activity and health outcomes. *NEJM Evid.* **1**, EVIDra2200190 10.1056/EVIDra2200190 (2022). 38319830

[7] Nagappan, A., Krasniansky, A. & Knowles, M. Patterns of ownership and usage of wearable devices in the United States, 2020-2022: survey study. *J. Med. Internet Res.* **26**, e56504 10.2196/56504 (2024). PMC11316147 39058548

[8] Mobile Fact Sheet. Pew Research Center. Updated November 13 2024. (Mobile Fact Sheet, 2025).

[9] Denny, J. C. et al. The “All of Us” research program. *N. Engl. J. Med.* **381**, 668–676 10.1056/NEJMsr1809937 (2019). PMC8291101 31412182

[10] Denny, J. C. et al. PheWAS: demonstrating the feasibility of a phenome-wide scan to discover gene–disease associations. *Bioinformatics* **26**, 1205–1210 10.1093/bioinformatics/btq126 (2010). PMC2859132 20335276

[11] Bastarache, L. Using phecodes for research with the electronic health record: from PheWAS to PheRS. *Annu. Rev. Biomed. Data Sci.* **4**, 1–19 10.1146/annurev-biodatasci-122320-112352 (2021). PMC9307256 34465180

[12] Shuey, M. M. et al. Next-generation phenotyping: introducing phecodeX for enhanced discovery research in medical phenomics. *Bioinformatics*.39 10.1093/bioinformatics/btad655(2023). PMC10627409 37930895

[13] Barker, J. et al. Physical activity of UK adults with chronic disease: cross-sectional analysis of accelerometer-measured physical activity in 96 706 UK Biobank participants. *Int. J. Epidemiol.* **48**, 1167–1174 10.1093/ije/dyy294 (2019). PMC6693885 30721947

[14] Chen, Z. et al. Daily heart rate per step: a wearables metric associated with cardiovascular disease in a cross sectional study of the All of Us Research Program. *J. Am. Heart Assoc.* **14**, e036801 10.1161/JAHA.124.036801 (2025). PMC12184247 40156587

[15] Ke, Y. et al. Phenome-wide association of physical activity with morbidity and mortality risk in China: a prospective cohort study. * Innovation* **6**, 100886 10.1016/j.xinn.2025.100886 (2025). PMC12277735 40697781

[16] Master, H. et al. Association of step counts over time with the risk of chronic disease in the All of Us Research Program. *Nat. Med.* **28**, 2301–2308 10.1038/s41591-022-02012-w (2022). PMC9671804 36216933

[17] Watts, E. L. et al. Association of accelerometer-measured physical activity level with risks of hospitalization for 25 common health conditions in UK adults. *JAMA Netw. Open* **6**, e2256186–e2256186 10.1001/jamanetworkopen.2022.56186 (2023). PMC9936337 36795414

[18] Khurshid, S. et al. Wearable accelerometer-derived physical activity and incident disease. *npj Digit. Med.* **5**, 131 10.1038/s41746-022-00676-9 (2022). PMC9440134 36056190

[19] Physical Activity Guidelines for Americans https://odphp.health.gov/sites/default/files/2019-09/Physical_Activity_Guidelines_2nd_edition.pdf (2018).

[20] Hutcheon, J. A., Chiolero, A. & Hanley, J. A. Random measurement error and regression dilution bias. *BMJ* **340** c2289, 10.1136/bmj.c2289 (2010). 20573762

[21] Hilden, P., Schwartz, J. E., Pascual, C., Diaz, K. M. & Goldsmith, J. How many days are needed? Measurement reliability of wearable device data to assess physical activity. *PLoS ONE* **18**, e0282162 10.1371/journal.pone.0282162 (2023). PMC9956594 36827427

[22] Togo, F. et al. How many days of pedometer use predict the annual activity of the elderly reliably? *Med. Sci. Sports Exercise* **40**, 1058–1064 (2008). 10.1249/MSS.0b013e318167469a 18461001

[23] Zisou C., et al. Reproducibility and associated regression dilution bias of accelerometer-derived physical activity and sleep in the UK Biobank. *medRxiv*. 2025:2025.01.16.25320679 10.1101/2025.01.16.25320679 41718664

[24] Small, S. R. et al. Self-supervised machine learning to characterise step counts from wrist-worn accelerometers in the UK Biobank. *Med Sci. Sports Exerc*10.1249/mss.0000000000003478 (2024). PMC11402590 38768076

[25] Strain, T. et al. Impact of follow-up time and analytical approaches to account for reverse causality on the association between physical activity and health outcomes in UK Biobank. *International Journal of Epidemiology* **49**, 162–172 10.1093/ije/dyz212 (2020). PMC7124507 31651957

[26] Bailey C. P. et al. Fitbit physical activity and sleep data in the All of Us Research Program: data exploration and processing considerations for research. *Med. Sci. Sports Exercise*. **1249**, 3804 (2025) 10.1249/mss.0000000000003804 PMC12264798 40605186

[27] Stamatakis, E. et al. Association of wearable device-measured vigorous intermittent lifestyle physical activity with mortality. *Nat. Med.* **28**, 2521–2529 10.1038/s41591-022-02100-x (2022). PMC9800274 36482104

[28] Tran, T. C. et al. PheWAS analysis on large-scale biobank data with PheTK. *Bioinformatics*. 2024;41 10.1093/bioinformatics/btae719 PMC11709244 39657951

[29] von Elm, E. et al. The strengthening the reporting of observational studies in epidemiology (STROBE) statement: guidelines for reporting observational studies. *PLoS Med.* **4**, e296 10.1371/journal.pmed.0040296 (2007). PMC2020495 17941714

