# Supplementary material for: 11 million days of longitudinal Fitbit data reveal novel future health insights
Source: Nat Commun. 2026 Oct 14;17:11492. doi: 10.1038/s41467-026-78529-2 (PMC13743742; doi:10.1038/s41467-026-78529-2)
Supplement: Supplementary file 2 [file 41467_2026_78529_MOESM2_ESM.pdf]

Supplementary Data

Title: Supplementary Data 1

Description: PheWAS summary statistics across all four wearable-derived activity metrics (step count, peak 1-minute cadence, peak 30-minute cadence, and daily heart rate per step [DHRPS]), from both Cox (incident) and logistic (prevalent) regression models. Results are organized into five worksheets, one per time window (Day, Week, Month, Six months, Year), each listing every tested phecode with its hazard ratio (HR) or odds ratio (OR) and 95% confidence interval. HR and OR values that reach Bonferroni-corrected significance ($\alpha = 0.05$ divided by the number of phecodes tested within that time window) are bolded.

Title: Supplementary Data 2

Description: Summary of which activity metrics and time windows each phecode reached Bonferroni-corrected significance in, split by regression model. Results are organized into eight worksheets, one per combination of model (logistic, Cox) and activity metric (step count, peak 1-minute cadence, peak 30-minute cadence, DHRPS). Each sheet lists all phecodes that were Bonferroni-significant in at least one time window, with an "X" marking the specific time windows (Day, Week, Month, Six months, Year) in which significance was reached.
