# Supplementary material for: 11 million days of longitudinal Fitbit data reveal novel future health insights
Source: Nat Commun. 2026 Oct 14;17:11492. doi: 10.1038/s41467-026-78529-2 (PMC13743742; doi:10.1038/s41467-026-78529-2)
Supplement: Supplementary file 3 [file 41467_2026_78529_MOESM5_ESM.pdf]

nature portfolio

Corresponding author(s): Evelynne Fulda
Last updated by author(s): Jun 3, 2026

Reporting Summary

Nature Portfolio wishes to improve the reproducibility of the work that we publish. This form provides structure for consistency and transparency in reporting. For further information on Nature Portfolio policies, see our Editorial Policies and the Editorial Policy Checklist.

Statistics

For all statistical analyses, confirm that the following items are present in the figure legend, table legend, main text, or Methods section.

n/a	Confirmed	
☐	☒	The exact sample size (*n*) for each experimental group/condition, given as a discrete number and unit of measurement
☐	☒	A statement on whether measurements were taken from distinct samples or whether the same sample was measured repeatedly
☐	☒	The statistical test(s) used AND whether they are one- or two-sided<br>*Only common tests should be described solely by name; describe more complex techniques in the Methods section.*
☐	☒	A description of all covariates tested
☐	☒	A description of any assumptions or corrections, such as tests of normality and adjustment for multiple comparisons
☐	☒	A full description of the statistical parameters including central tendency (e.g. means) or other basic estimates (e.g. regression coefficient) AND variation (e.g. standard deviation) or associated estimates of uncertainty (e.g. confidence intervals)
☐	☒	For null hypothesis testing, the test statistic (e.g. *F*, *t*, *r*) with confidence intervals, effect sizes, degrees of freedom and *P* value noted<br>*Give P values as exact values whenever suitable.*
☒	☐	For Bayesian analysis, information on the choice of priors and Markov chain Monte Carlo settings
☒	☐	For hierarchical and complex designs, identification of the appropriate level for tests and full reporting of outcomes
☐	☒	Estimates of effect sizes (e.g. Cohen's *d*, Pearson's *r*), indicating how they were calculated

Our web collection on statistics for biologists contains articles on many of the points above.

Software and code

Policy information about availability of computer code

Data collection	Data were from the All of Us Research Program and are available to all approved researchers on the All of Us Researcher Workbench.
Data analysis	All analyses were conducted using R and were performed within the All of Us Researcher Workbench. Analysis notebooks are available to any approved All of Us controlled tier researchers. A GitHub repository with code can be found at: https://github.com/efulda/aou-11-million-days-fitbit

For manuscripts utilizing custom algorithms or software that are central to the research but not yet described in published literature, software must be made available to editors and reviewers. We strongly encourage code deposition in a community repository (e.g. GitHub). See the Nature Portfolio guidelines for submitting code & software for further information.

Data

Policy information about availability of data

All manuscripts must include a data availability statement. This statement should provide the following information, where applicable:
- Accession codes, unique identifiers, or web links for publicly available datasets
- A description of any restrictions on data availability
- For clinical datasets or third party data, please ensure that the statement adheres to our policy

Data were from the All of Us Research Program and are available to all approved researchers on the All of Us Researcher Workbench. Researchers can register for access at www.researchallofus.org

Research involving human participants, their data, or biological material

Policy information about studies with human participants or human data. See also policy information about sex, gender (identity/presentation), and sexual orientation and race, ethnicity and racism.

Reporting on sex and gender	Sex at birth was assessed via self-report. This variable was used as disease risk can vary by sex, and some conditions occur exclusively in males or females due to biological differences.
Reporting on race, ethnicity, or other socially relevant groupings	Analyses adjusted for age, sex at birth (self-reported), race/ethnicity (self-reported), alcohol consumption (self-reported), smoking history (self-reported), annual income (self-reported), and whether a participant owned a Fitbit or was provided one by the study.
Population characteristics	The median age was 57.3 (interquartile range: 42.3, 69.3) years. Over two thirds of participants were female (69%), the majority were current alcohol drinkers (81%), and more than one third of participants were current/past smokers (38%).
Recruitment	Data were obtained from the All of Us Research Program. Recruitment procedures were outside the scope of the current analysis; however, participants enrolled either by signing up directly online or through a participating health care provider organization.
Ethics oversight	The All of Us Research Program (protocol number: 2021-02-TN-001) was approved by the All of Us Institutional Review Board.

Note that full information on the approval of the study protocol must also be provided in the manuscript.

Field-specific reporting

Please select the one below that is the best fit for your research. If you are not sure, read the appropriate sections before making your selection.

[x] Life sciences [ ] Behavioural & social sciences [ ] Ecological, evolutionary & environmental sciences

For a reference copy of the document with all sections, see nature.com/documents/nr-reporting-summary-flat.pdf

Life sciences study design

All studies must disclose on these points even when the disclosure is negative.

Sample size	Included participants 1) had at least one valid day of Fitbit data for all four metrics, 2) linked their electronic health record data, 3) self-reported their sex at birth at the baseline All of Us study visit.
Data exclusions	Daily Fitbit values were excluded if they were invalid (valid days were those with at least 16 hours of wear time and with values in a feasible range [step count >100 and <75,000; cadence <210 steps/min; DHRPS <3 standard deviations above the overall mean]).
Replication	A key aim of the study was to utilize and compare multiple methods to assess observed associations between device-measured physical activity and a range of disease outcomes, by looking at 1) multiple exposure metrics, 2) different exposure durations, 3) both prevalent and incident outcomes, and 4) multiple sensitivity analyses. There is currently no comparable data source with this duration of wearable accelerometer data linked to electronic health record data.
Randomization	This analysis used data from a national cohort study and thus randomization was not part of the study design. Further, randomization is not feasible for a lifetime behavioral phenotype such as physical activity. However, we adjusted for many potential confounders (as listed above) and conducted additional sensitivity analyses.
Blinding	This analysis used data from a national cohort study and therefore participants were not allocated to groups and blinding was not necessary.

Reporting for specific materials, systems and methods

We require information from authors about some types of materials, experimental systems and methods used in many studies. Here, indicate whether each material, system or method listed is relevant to your study. If you are not sure if a list item applies to your research, read the appropriate section before selecting a response.

Materials & experimental systems

n/a	Involved in the study
☒	☐ Antibodies
☒	☐ Eukaryotic cell lines
☒	☐ Palaeontology and archaeology
☒	☐ Animals and other organisms
☒	☐ Clinical data
☒	☐ Dual use research of concern
☒	☐ Plants

Methods

n/a	Involved in the study
☒	☐ ChIP-seq
☒	☐ Flow cytometry
☒	☐ MRI-based neuroimaging

Plants

Seed stocks

Report on the source of all seed stocks or other plant material used. If applicable, state the seed stock centre and catalogue number. If plant specimens were collected from the field, describe the collection location, date and sampling procedures.

Novel plant genotypes

Describe the methods by which all novel plant genotypes were produced. This includes those generated by transgenic approaches, gene editing, chemical/radiation-based mutagenesis and hybridization. For transgenic lines, describe the transformation method, the number of independent lines analyzed and the generation upon which experiments were performed. For gene-edited lines, describe the editor used, the endogenous sequence targeted for editing, the targeting guide RNA sequence (if applicable) and how the editor was applied.

Authentication

Describe any authentication procedures for each seed stock used or novel genotype generated. Describe any experiments used to assess the effect of a mutation and, where applicable, how potential secondary effects (e.g. second site T-DNA insertions, mosiacism, off-target gene editing) were examined.
