# Supplementary material for: 11 million days of longitudinal Fitbit data reveal novel future health insights
Source: Nat Commun. 2026 Oct 14;17:11492. doi: 10.1038/s41467-026-78529-2 (PMC13743742; doi:10.1038/s41467-026-78529-2)
Supplement: Supplementary file 4 [file 41467_2026_78529_MOESM6_ESM.pdf]

nature portfolio

Peer Review File

11 million days of longitudinal Fitbit data reveal novel future health insights

Corresponding Author: Ms Evelynne Fulda

This file contains all reviewer reports in order by version, followed by all author rebuttals in order by version.

Version 0:

Reviewer comments:

Reviewer #1

(Remarks to the Author)
Thank you for the careful and thorough revisions to this manuscript. The updates represent a meaningful improvement, and I appreciate the authors' attention to the concerns raised. I am largely satisfied with the direction of the revisions pending a few final clarifications.

I have three remaining comments for the authors to address:

1. (Previously R1, Comment 10) The revised language — "stronger associations with greater effect sizes for disease risk" — is an improvement, but the statement remains somewhat vague. If this is primarily a visual observation that associations appear greater with longer assessment periods, the language should reflect that modesty rather than implying a statistically supported conclusion.

2. (Previously R1, Comment 17) I appreciate the addition of the following clarification in the Methods (page 23, lines 371-373): "Phecodes are hierarchical: a parent phecode includes all of its descendants. For example, the phecode for Type 2 diabetes covers conditions such as Type 2 diabetes mellitus with ketoacidosis. However, the reverse is not true." While this is helpful context, I still remain uncertain how a reader should interpret the findings. Could the authors please directly answer the following questions and consider making this more direct in the paper: Were similar or overlapping outcomes combined or accounted for in terms of overlap? Could participants be double-counted across related outcomes? Do these associations reflect truly unique conditions per participant, or does a single participant could contribute to multiple related outcomes?

3. (New comment) In Supplementary Figures 9, which compare incident and prevalent associations with physical activity metrics, the legend does not specify which time window of accelerometer data is used. Please add a clear notation indicating the time window (e.g., 1 day, 1 week, 1 month, etc.) so that readers can properly interpret and contextualize these figures.

Thank you again for your efforts on this revision.

Reviewer #2

(Remarks to the Author)
Thank you to the authors for addressing my previous comments thoughtfully. The manuscript is improved.

However, I am just not convinced that etiological insights are possible with with only 2-3 years of prospective data. Such insights cannot really be supported by prevalent cases, so the prospective data is key here. The sophistication and rigor of the data analysis cannot overcome this fundamental limitation.

I would expect the limitation of the short follow-up time to be clearly articulated in the Discussion with a reference to this article https://doi.org/10.1093/ije/dyz212

Reviewer #3

(Remarks to the Author)

Open Access This Peer Review File is licensed under a Creative Commons Attribution 4.0 International License, which permits use, sharing, adaptation, distribution and reproduction in any medium or format, as long as you give appropriate credit to the original author(s) and the source, provide a link to the Creative Commons license, and indicate if changes were made.
In cases where reviewers are anonymous, credit should be given to 'Anonymous Referee' and the source.
The images or other third party material in this Peer Review File are included in the article's Creative Commons license, unless indicated otherwise in a credit line to the material. If material is not included in the article's Creative Commons license and your intended use is not permitted by statutory regulation or exceeds the permitted use, you will need to obtain permission directly from the copyright holder.
To view a copy of this license, visit https://creativecommons.org/licenses/by/4.0/

NCOMMS-26-017865A
Response to Reviewers

We thank both the reviewers and the editorial team for their feedback. We have addressed each of the suggestions below (in orange text) and have revised our manuscript accordingly.

In addition to addressing the Reviewer comments below, we discovered that adding a requirement of wear time using heart rate improved results. The message is the same. More associations were found across all activity phenotypes. Most notably, this improved the stability of the peak 1-min cadence metric. All analyses have been rerun and double checked. We have added this additional data cleaning aspect to the methods and believe this strengthens the paper toward a reproducible method for analyzing real world activity monitoring data.

Reviewer #1 (Remarks to the Author):

Thank you for the careful and thorough revisions to this manuscript. The updates represent a meaningful improvement, and I appreciate the authors' attention to the concerns raised. I am largely satisfied with the direction of the revisions pending a few final clarifications.

I have three remaining comments for the authors to address:

R1, Comment 1: (Previously R1, Comment 10) The revised language — "stronger associations with greater effect sizes for disease risk" — is an improvement, but the statement remains somewhat vague. If this is primarily a visual observation that associations appear greater with longer assessment periods, the language should reflect that modesty rather than implying a statistically supported conclusion.

Author response: We appreciate this comment and the opportunity to clarify. We confirm that this observation is statistically supported. Differences in associations across time windows were formally tested and compared using a z-score approach. We have revised the manuscript to make this explicit. The revised text now reads (page 11, lines 191-195):

> "Second, although all time windows yielded informative associations, longer assessment periods were generally associated with greater effect sizes for disease risk, with differences across time windows confirmed statistically using z-score comparisons. Counts of statistically significant associations for each activity phenotype broadly plateaued at around 6 months."

R1, Comment 2: (Previously R1, Comment 17) I appreciate the addition of the following clarification in the Methods (page 23, lines 371-373): "Phecodes are hierarchical: a parent phecode includes all of its descendants. For example, the phecode for Type 2 diabetes covers conditions such as Type 2 diabetes mellitus with ketoacidosis. However, the reverse is not true." While this is helpful context, I still remain uncertain how a reader

should interpret the findings. Could the authors please directly answer the following questions and consider making this more direct in the paper:

Were similar or overlapping outcomes combined or accounted for in terms of overlap?

Author response: No, similar or overlapping outcomes were not combined. Each phecode was analyzed as a separate outcome. Because phecodes are hierarchical, a parent phecode's case definition includes all cases from its child phecodes. For example, a participant who is a case in the Type 2 diabetes (phecode: EM_202.2) analysis would also be a case in the parent Diabetes mellitus (phecode: EM_202) analysis.

Could participants be double-counted across related outcomes?

Author response: Within any single phecode's analysis, each participant is counted only once as a case or control. However, because of the hierarchical structure, the same participant can appear as a case in more than one phecode-level analysis (e.g., in both the Type 2 diabetes and Diabetes mellitus analyses). So there is no double-counting within a given outcome's analysis, but overlap of participants across related, hierarchically linked outcomes is expected.

Do these associations reflect truly unique conditions per participant, or does a single participant could contribute to multiple related outcomes?

Author response: A single participant can contribute to multiple related outcomes when those outcomes are hierarchically linked. As such, findings for closely related phecodes should not be interpreted as fully independent signals.

This is a standard approach for phecode-based analyses and has been widely used in PheWAS studies since the release of the phecode terminology in 2013. Importantly, we account for the large number of associations tested using a conservative Bonferroni correction. We used a validated Python package (PheTK) to run this analysis.

We appreciate the opportunity to clarify this point. The revised text now reads (page 17-18, lines 341-351):

> "Each phecode was analyzed as a separate outcome. Phecodes are hierarchical: most phecodes have no child phecodes, but some do, and a parent phecode's case definition includes all of its descendant phecodes. Because of this hierarchical structure, a single participant can contribute to multiple related outcomes: for example, a participant who is a case for Type 2 diabetes (phecode: EM_202.2) would also be a case for the parent phecode Diabetes mellitus (phecode: EM_202). Associations across hierarchically related phecodes are therefore not statistically independent, and findings across related conditions should be interpreted as potentially reflecting the same underlying signal at different levels of diagnostic specificity. Our Bonferroni correction for multiple testing remains conservative given this overlap."

R1, Comment 3: (New comment) In Supplementary Figures 9, which compare incident and prevalent associations with physical activity metrics, the legend does not specify which time window of accelerometer data is used. Please add a clear notation indicating the time window (e.g., 1 day, 1 week, 1 month, etc.) so that readers can properly interpret and contextualize these figures. Thank you again for your efforts on this revision.

Author response: We have updated the legend of Supplementary Figure 9 to explicitly state the accelerometer time window used (year) so that readers can properly interpret and contextualize these findings.

> "Each point represents a phecode analyzed in both logistic regression (prevalent cases, y-axis) and Cox regression (incident cases, x-axis), with effect sizes shown as odds ratios (OR) and hazard ratios (HR) per (a) 10 steps/min higher, (b) 10 steps/min higher, or (c) 0.01 DHRPS higher from the 1-year accelerometer assessment. The dashed diagonal indicates equality of HR and OR."

Reviewer #2 (Remarks to the Author):

Thank you to the authors for addressing my previous comments thoughtfully. The manuscript is improved. However, I am just not convinced that etiological insights are possible with with only 2-3 years of prospective data. Such insights cannot really be supported by prevalent cases, so the prospective data is key here. The sophistication and rigor of the data analysis cannot overcome this fundamental limitation. I would expect the limitation of the short follow-up time to be clearly articulated in the Discussion with a reference to this article https://doi.org/10.1093/ije/dyz212

Author response: We thank the reviewer for this feedback and agree that it warrants direct acknowledgment. Strain et al. (2020, Int J Epidemiol) demonstrate clearly that short follow-up periods in large cohort studies can produce associations susceptible to reverse causation and may not be sufficient to support etiological conclusions. We have added the following to the Limitations section of the Discussion (page 14, lines 268-276):

> "Although prospective analyses were performed, the median follow-up period was relatively short for many chronic diseases. Consequently, these findings should be interpreted as evidence of prospective associations or early biomarkers rather than definitive etiological relationships, given the susceptibility to reverse causation, whereby pre-existing subclinical disease may influence physical activity behavior rather than the reverse (Strain et al., 2020). Whilst a sensitivity analysis excluding events from the first three years of follow-up yielded broadly consistent findings, longer-term studies will be required to establish temporal and potentially causal pathways."
